# Problematic usage of the internet among Hungarian elementary school children: a cross-sectional study

**DOI:** 10.1186/s12889-024-18593-9

**Published:** 2024-04-17

**Authors:** Adam Szapary, Gergely Feher, Ildiko Radvanyi, Eva Fejes, Gabor Daniel Nagy, Csaba Jancsak, Lilla Horvath, Zoltan Banko, Gyula Berke, Krisztian Kapus

**Affiliations:** 1 Centre for Occupational Medicine, Medical School, University of Pécs, Nyár u. 8, Pécs, 7624 Hungary; 2 Department of Primary Health Care, University of Pécs, Pécs, 7623 Hungary; 3 Interdisciplinary R&D and Innovation Center of Excellence, Social Responsibility Competence Centre, University of Szeged, Szeged, 6720 Hungary; 4 Hospital of Komló, Komló, 7300 Hungary; 5 JGYPK Department of Applied Social Sciences, University of Szeged, Szeged, 6720 Hungary; 6 Department of Labour Law and Social Security Law, Faculty of Law, University of Pécs, Pécs, 7622 Hungary

**Keywords:** Problematic internet use, Student, Cross-sectional study, Questionnaire, Epidemiology, Risk factors

## Abstract

**Introduction:**

Problematic usage of the internet (PUI) is perhaps one of the most frequently studied phenomena of the 21st century receiving increasing attention in both scientific literature and the media. Despite intensive research there have been relatively few meaningful studies among elementary school students in Hungary and worldwide, who may be considered as a high-risk population with regard to problematic internet use. The aim of our study was to carry out a complex research focusing on the prevalence and risk factors of PUI among elementary school children aged 10–15 years (Grade 5–8).

**Methods:**

Demographics included were gender, age, place of stay, type of residence, family type, parental education, start of internet use, used devices, daily internet use, purpose of internet use, internet accounts, ways of keeping in touch with friends and sporting activities. PUI was evaluated using the paper-based version of the Potentially Problematic Use of the Internet Questionnaire.

**Results:**

Overall, 2000 paper-based questionnaires were successfully delivered and the final analysis included 1168 responses (overall response rate 58.4%). Mean age was 12.55 ± 1.24 years. Female gender (OR = 2.760, *p* = 0,006, CI 95% 0.065 to 0.384), younger age (11–12 years) (OR = 3.812, *p* < 0.001, 95% CI: 1.747–4.731), early exposure to the internet (OR = 3.466, *p* = 0.001, 95% CI 1.535–5.446), living in a small village (OR = 1.081, *p* = 0.002, 95% CI 1.041–1.186) urgency to answer online (OR = 4.677, *p* < 0.001, 95% CI: 2.714–6.639), decreased frequency of personal contact with friends (OR = 2.897, *p* = 0.004, 95% CI: 1.037–1.681), spending more than 6 h online (OR = 12.913, *p* < 0.001, 95% CI: 10.798–14.892), morning and nighttime internet use (OR = 3.846, *p* < 0.001, CI 95% 1.886–5.810) and never doing any sports (OR = 2.016, *p* = 0.044, 95% CI: 1.050–3.354) were independently associated with problematic internet use.

**Conclusions:**

Based on the results of our questionnaire survey more than 10% seemed to be problematic users in our study population, which is a relatively high rate. Early exposure to the internet as well as younger age were strongly related to this phenomenon. Duration of being online as well as daily time interval of internet use are important predisposing factors. Scarcely studied social factors such as being online at the expense of personal relationships and the lack of physical activity should be payed more attention to prevent the development of PUI.

## Introduction

Problemtic usage of the internet is perhaps one of the most frequently studied phenomena of the 21st century receiving more and more attention in both scientific literature and media. Digitization has become an integral part of our everyday life (including work and private life) due to the widespread availability of affordable and fast broadband internet connection [1]. This technological development has fundamentally changed our lives in recent decades mostly affecting the younger generations. In addition to the positive effects of digitization such as the rapid flow of information or the widespread availability of human knowledge databases, it inevitably has its downsides such as problematic internet use, which shows similar patterns to classic addiction behaviours (alcohol or drug abuse). Digital device use has become an integral part of our everyday life, consequently, it is difficult to draw the line between normal and problematic internet use. As being online is a widely accepted activity and is generally thought to be harmless, dependence is hard to recognize for both the individual and their environment [2, 3].

Despite the above mentioned intensive research carried out on the topic, compulsive internet use is still not classified as a medical condition (apart from problematic gaming), but is labeled as a phenomenon. As PUI (or so called internet addiction) is not properly classified, there is no generally accepted and validated assessment tool for its detection. There are several widely used questionnaires, but these are mainly developed for research and not for clinical use [4, 5]. It is generally agreed that problematic or compulsive users spend most of their lives in front of a screen and being offline they continue to think and dream about online activities, which negatively affects their private and social lives similar to substance abuse [6].

PUI may affect up to 7% of the total population, however, there are significant epidemiological and geographical differences [2]. Prevalence is higher among younger users and individuals from East Asia, with a rate of 20% or higher [2, 6]. The most important risk factors include early use of digital devices/internet use, as early exposure can be associated with a higher risk of developing PUI. Male sex seems to be associated with a two to five times higher addiction rate compared to females, which can be explained by differences in personality traits such as lower self-control, higher levels of impulsivity, novelty-seeking tendencies, however, results are conflicting [7–10].

Proper family functioning is fundamental in the prevention of the development of PUI. The lack of a supportive environment, more superficial or inappropriate parent-child relationships (especially neglect or potential abuse) are all important risk factors. Interestingly, a positive father figure seems to be an important protective factor [11, 12]. Turning to online activities is often a consequence of trying to escape from real-world conflicts [11, 12]. The rate of compulsive users is higher among those living in rural areas and those of low socio-economic status [13].

It seems that certain individual personality traits carry a higher risk of PUI such as impulsivity, aggression and hostility [14]. Neurosis, that is the tendency to nervousness and worry can also be a potential predisposing factor similar to withdrawal (lack of social interactions and perceived or real offline failure) [15, 16]. The duration of online activities should be taken into consideration especially for first-time users, it is strongly correlated with later problematic use. The purpose of being online is also an important predictor of problematic usage, for example work and gaming carry different risk stratifications [10, 17–19]. Recent studies have showed that ‘time wasters’ or watching streams can enhance PUI among older individuals (> 40 years) [17–19]. Night time internet use (at the expense of sleep) also can also be a potential sign of problematic usage [9].

PUI is linked to weight problems as both obesity (sedentary lifestyle, eating fast food, etc.) and malnutrition (being online instead of eating or having a false body image) can develop [20]. Recent studies have showed a possible link between PUI and sympathetic hyperactivity which may lead to the development of high blood pressure and cardiovascular diseases [21, 22]. There seems to be a close association with diabetes, bone and musculoskeletal pain among adults, which is probably linked to sedentary lifestyle, developing unhealthy postures and eating habits [21, 22]. Based on cross-sectional studies PUI is strongly linked to several mental disorders and illnesses including anxiety, depression, attention deficit hyperactivity disorder, autism as well as to other addictions such as alcohol or drug abuse, however, the causality is not entirely clarified [23, 24].

Despite intensive research there have been relatively few meaningful studies among elementary school students who may be considered a high-risk population [25]. A recent meta-analysis included a total of 20 studies, among which only 8 contained more than 1000 participants with significant methodological differences [26]. On the other hand, there are only few proper questionnaires, furthermore, the widely used validated questionnaires developed so far have caused several difficulties in this population (some questions which may be easily understandable for adults may pose difficulties for younger particpants for example and several questions may prove irrelevant etc.). The Potentially Problematic Use of the Internet Questionnaire developed by Kaltiala et al. was designed to target this population (aged 10–18) and has been used successfully in recent research studies [27, 28].

The aim of our study was to carry out a complex research focusing on the prevalence and risk factors of PUI among elementary school children aged 10–15 years (Grade 5–8). We included the most important risk factors (as seen above) into our analysis such as detailed demographics (sex, age, place of stay, type of residence, family type, parental education), and habits of internet use (onset of internet use, applied devices, daily internet use, purpose of internet use, number of accounts), furthermore the role of ways of keeping in touch with friends and sporting activities (which are rarely studied) were also recorded.

## Materials and methods

This cross-sectional, paper-based questionnaire study applying a non-probability sampling method was conducted in 10 large educational sites in Centre Hungary between September 2022 and February 2023 (see Acknowledgement section) The study was approved by the Hungarian Medical Research Council (BMEÜ/1794-3/2022/EKU). Prior to questionnaire distribution consent was obtained from all school authorities. Informed consents were also signed by participants and their parents/guardians before entering the survey. Inclusion criteria comprised willingness to participate and active student status during the study period (and having signed an informed consent as mentioned above).

Demographic parameters included were gender, age, place of stay, type of residence, type of family (main caregiver), parents’ educational achievement, age at onset of digital device use. Details about daily time of internet use, daily time interval, type of used devices, purpose of being online and number of online accounts were also recorded. Data about always being online (never logging out), urgency to answer (an inner urge to immedately respond to incoming messages), ways of keeping in touch with friends (online vs. offline) and sporting activities were also collected.

Problematic usage of the internet was evaluated by the questionnaire originally developed by Kaltiala-Heino et al. [27]. This survey contains 7 items, all of them are evaluated on a 5-point Likert scale from 1 to 5 (from never to always). This questionnaire was developed according to the DSM-IV criteria of pathological gambling omitting 3 questions out of ten as not relevant for this age group [27, 28]. According to the authors’ methodology problematic internet use was detected if at least four out of the seven criteria applied in this study were met (indicating “often” or “always”, i.e. reaching 4 or 5 points in 4 categories out of 7) [27].

As this questionnaire was not available in Hungarian we asked the corresponding author to provide the original questionnaire for linguistic validation and cultural adaptation according to the current linguistic validation protocols [29, 30]. After having received the permission and the survey, two official translators created a Hungarian translation. In the next step, a medical professional was also invited and the joint committee released the first version. This was followed by the jointly created back translation of the Hungarian version with the help of two native English speakers who had a native-like command of Hungarian. In the final step, an expert group containing two neurologists, a linguist and an applied linguist created the final version. Cronbach’s alpha for the scale was 0.56 for girls and 0.66 for boys in the case of the original questionnaire, while it was 0.807 for this Hungarian version [27].

Based on the results of the Potentially Problematic Use of the Internet Questionnaire, participants were categorized as [1] problematic internet users or [2] normal users. Data were evaluated as mean ± SD (standard deviation) by Student’s *t*-test or chi-square test to detect significant differences among examined parameters (demographical data, habits of internet use, keeping in touch with friends and sporting activities). To clarify the role of different parameters as independent risk factors for problematic internet use, logistic regression analysis was carried out including all the examined parameters found to be significant in an univariate analysis (see above). For all odds ratios, an exact confidence interval (CI) of 95% was constructed. Data analysis was performed using SPSS (version 11.0, IBM, New York, NY, USA).

## Results

Overall 2000 questionnaires were successfully delivered and 1367 responses were received yielding a response rate of 68.35%. Among these 199 were partially completed, therefore, the final analysis contained 1168 responses (overall response rate 58.4%). The mean age of the study population was 12.55 ± 1.24 years. Overall, the study group consisted of 595 boys (50.9%, mean age 12.5 ± 1.23 years) and 573 girls (49.1%, mean age 12.6 ± 1.28 years). According to Kolmogorov-Smirnov and Shapiro-Wilk test (*p* = 0.206, *p* = 0.091) the distribution is normal. Detailed demographics can be seen in Table 1.

**Table 1:** Demograpic parameteres and their relation to internet addiction in the study population (*p < 0.05; **p < 0.001)

	Whole population 1168 (100%)	Normal users 1041 (100%)	Problematic users 127 (100%)
**Sex (X** ^**2**^ ** (1) = 2.094)**			
males	595 (50.9%)	538 (51.7%)	57 (44.9%)
females	573 (49.1%)	503 (48.3%)	70 (55.1%)*
**Age (X**^**2**^ (7) **= 8.293)**			
9–10	51 (4.3%)	46 (4.4%)	5 (3.9%)
11–12	511 (43.8%)	442 (42.5%)	69 (54.3%)*
13–14	562 (48.1%)	513 (49.3%)	49 (38.7%)
15–16	44 (3.8%)	40 (3.8%)	4 (3.1%)
**Place of stay (X**^**2**^ (5) **= 7.904)**			
capital	174 (14.9%)	151 (14.5%)	23 (18.1%)
big town/city	155 (13.3%)	147 (14.1%)	8 (6.3%)
small town	492 (42.1%)	440 (42.3%)	52 (40.9%)
large village	293 (25.1%)	257 (24.7%)	36 (28.4%)
small village	54 (4.6%)	46 (4.4%)	8 (6.3%)*
**Type of residence (X**^**2**^ (3) **= 0.249)**			
farm	59 (5.1%)	52 (5.1%)	7 (5.5%)
flat	276 (23.6%)	247 (23.7%)	29 (22.8%)
house	833 (71.3%)	742 (71.3%)	91 (71.7%)
**Family type (main caregiver) (X**^**2**^ (5) **= 0.559)**			
two parents	911 (78%)	811 (77.9%)	100 (78.7%)
single parent	178 (15.2%)	158 (15.2%)	20 (15.7%)
grandparents	13 (1.1%)	11 (1.1%)	2 (1.6%)
foster parents	9 (0.8%)	8 (0.7%)	1 (0.8%)
other	57 (4.9%)	53 (5.1%)	4 (3.2%)
**Father’s educational achievement (X**^**2**^ (6) **= 25.625)**			
no graduation	20 (1.7%)	15 (1.4%)	5 (3.9%)
primary school	95 (8.1%)	79 (7.6%)	16 (12.6%)
vocational school	218 (18.7%)	199 (19.2%)	19 (15%))
high school	190 (16.2%)	172 (16.5%)	18 (14.2%)
college/university	245 (21%)	233 (22.4%)	12 (9.4%)
unknown	400 (34.3%)	343 (32.9%)	57 (44.9%) **
**Mother’s educational achievement (X**^**2**^ (6) **= 37.058)**			
no graduation	24 (2%)	20 (1.9%)	4 (3.2%)
primary school	102 (8.7%)	83 (8%)	19 (15%)
vocational school	155 (13.3%)	143 (13.7%)	12 (9.4%)
high school	198 (17%)	181 (17.4%)	17 (13.4%)
college/university	314 (26.9%)	299 (28.7%)	15 (11.8%)
unkown	375 (32.1%)	315 (30.3%)	60 (47.2%) **

The mean age at the onset of internet use was 7.61 ± 2.12 years. More than half of the study population (54.7%) was practically always online (never logging out from accounts) and 37.1% of respondents tended to answer immediately to any message received. Almost two-thirds of the participants (60.5%) were engaged in daily online chats with friends and 854 (73.2%) meet their friends every day. 445 students (38.1%) spent 2–3 h online a day mainly in the afternoon (92.3%). The preferred device for internet use was mobile phone for the vast majority of our study group (92.7%). The purposes of internet use were the following: chatting (81.8%), playing online games (75.8%) and watching videos (76.2%) (Table 2).

**Table 2:** Internet use patterns and their relation to internet addiction (*p < 0.05; **p < 0.001)

	Whole population 1168 (100%)	Normal users 1041 (100%)	Problematic users 127 (100%)
**Start of internet use (years) (X** ^**2**^ ** (13) = 34.874)**			
1–2	17 (1.5%)	12 (1.2%)	5 (3.9%)
3–4	66 (5.7%)	56 (5.4%)	10 (7.9%)
5–6	259 (22.1%)	219 (21%)	40 (31.5%) *
7–8	404 (34.6%)	362 (34.8%)	42 (33.1%)
9–10	355 (30.4%)	327 (31.4%)	28 (22%)
11–12	65 (5.5%)	63 (6%)	2 (1.6%)
13	2 (0.3%)	2 (0.2%)	0 (0%)
**Time spent online (X**^**2**^ (7) **= 67.799)**			
1 h	156 (13.3%)	152 (14.6%)	4 (3.2%) **
2 h	220 (18.8%)	211 (20.3%)	9 (7.1%) **
3 h	225 (19.3%)	207 (19.9%)	18 (14.2%)
4 h	191 (16.4%)	171 (16.4%)	20 (15.7%)
5 h	121 (10.4%)	105 (10.1%)	16 (12.1%)
6 h	85 (7.3%)	69 (6.6%)	16 (12.6%)
> 6 h	170 (14.5%)	126 (12.1%)	44 (34.6%)**
**Preferred digital device (multiple answers) (X**^**2**^ (15) **= 11.672)**			
mobile phone	1083 (92.7%)	966 (92.8%)	117 (92.1%)
tablet	199 (17%)	174 (16.7%)	25 (19.7%)
laptop	403 (34.5%)	357 (34.3%)	46 (36.2%)
computer	327 (28%)	295 (28.3%)	32 (25.2%)
**Preferred time interval of being online (multiple answers) (X**^**2**^ (9) **= 56.926)**			
morning	280 (24%)	227 (21,8%)	53 (41.7%)
afternoon	1078 (92.3%)	959 (92.1%)	119 (93.7%)
night	419 (35.9%)	347 (33.3%)	72 (56.7%)
**Purpose of internet use (multiple answers) (X**^**2**^ (38) **= 66.492)**			
gaming	885 (75.8%)	786 (75.5%)	99 (77.9%)
online chat	944 (81.8%)	844 (81.1%)	100 (78.7%)
video watching	890 (76.2%)	785 (75.4%)	105 (82.7%)
watching films	691 (59.2%)	606 (58.2%)	85 (66.9%)
learning	616 (52.7%)	564 (54.2%)	52 (40.9%)

The most popular accounts were Messenger (85.5%), Youtube (78.9%) and Facebook (76.8%). More than one-third of the participants (34.5%) did not have any sporting activities (Table 3).

PUI was assessed with the Kaltiala-Heino Questionnaire as mentioned above. The Cronbach alpha was 0.807 for this questionnaire. The prevalence of problematic users was 10.9% (127/1168, 95% 10.1–11.5%) in our study population.

Among demographic parameters problematic internet use was associated with female sex (55.1% vs. 44.9%, *p* = 0.09), younger age (11–12 years old 42.5% vs. 54.3% *p* = 0.027) and living in a small village (4.4% vs. 6.3%, *p* = 0.017). Absence of knowledge about parents’ educational achievement was associated with PUI (32.9 vs. 44.9% and 30.3 vs. 47.2%, *p* < 0.001 in both cases). There was significant association between PUI and early exposure to the internet (5–6 years of age) (21.0% vs. 31.5%, *p* < 0.001). Urgency to answer incoming messages (34.5% vs. 41.7%, *p* < 0.001), daily online chat with friends (59.5% vs. 68.5%, *p* = 0.018), decreased frequency of personal contacts with friends (4.7% vs. 9.4%, *p* = 0.003), never doing any sports (33.3% vs. 44.1%, *p* = 0,003) and spending more than 6 h online (12.1% vs. 34.6%, *p* = 0,000) were strongly associated with PUI.

Interestingly, being online less than 2 h was associated with lower rates of PUI in our study population (14.6 vs. 3.2% and 20.3 vs. 7.1%, *p* < 0.001 in both cases) as well as regular sporting activities (18.2 vs. 10.2%, *p* = 0.026) (Table 3). Morning (schooltime) and nighttime internet use also increased the rate of PUI (21.8% vs. 41.7%, *p* = 0.001, and 33.3% vs. 56.7% *p* < 0.001). Watching videos (Youtube, live streams) (75.4% vs. 82.7%, *p* = 0.041) and having multiple online accounts (TikTok, Youtube, Twitter) (78.1% vs. 85.0%, *p* = 0.004, 69.1% vs. 77.9%, *p* = 0,001, 15.9% vs. 29.1%, *p* = 0,002) were also predictors of problematic internet use.

PUI was associated with having more than one online account in chidren under and above 13 years (the minimum age specified on websites) (8.4 vs. 10.8% for 2 accounts, *p* = 0.003; 83 vs. 85.1% for three accounts, *p* = 0.031 in children < 13 years and 1.3 vs. 7.5% for two accounts *p* < 0.001; 90.6 vs. 95.5%, *p* = 0.018 for three accounts among children ≥ 13 years) (not shown).

**Table 3:** Social activities and their relation to problematic internet use (*p < 0.05)

	Whole population 1168 (100%)	Normal users 1041 (100%)	Problematic users 127 (100%)
**Always online (X**^**2**^ (2) **= 19.766)**	639 (54.7%)	546 (52.5%)	93 (26.8%)
**Urgency to answer (X**^**2**^ (2) **= 27.495)**	433 (37.1%)	359 (34.5%)	74 (41.7%)
**Online chat with friends (X**^**2**^ (5) **= 7.440)**			
every day	706 (60.5%)	619 (59.5%)	87 (68.5%)
several times a week	271 (23.2%)	248 (23.8%)	23 (18.1%)
once a week	76 (6.5%)	70 (6.7%)	6 (4.7%)
every two weeks	27 (2.3%)	27 (2.6%)	0 (0%)
less often	88 (7.5%)	77 (7.4%)	11 (8.7%)
**Personal meetings with friends (X**^**2**^ (5) **= 9.723)**			
every day	854 (73.2%)	759 (72.9%)	95 (74.8%)
several times a week	186 (15.9%)	173 (16.6%)	13 (10.3%)
once a week	49 (4.2%)	43 (4.1%)	6 (4.7%)
every two weeks	19 (1.6%)	18 (1.7%)	1 (0.8%)
less often	60 (5.1%)	48 (4.7%)	12 (9.4%)
**Online accounts (multiple answers) (X** ^**2**^ **(151) = 172.320)**			
Facebook	918 (78.6%)	818 (78.6%)	100 (78.7%)
Youtube	921 (78.9%)	813 (78.1%)	108 (85%)
TikTok	818 (70%)	719 (69.1%)	99 (77.9%)
Messenger	999 (85.5%)	889 (85.4%)	110 (86.6%)
Instagram	683 (58.5%)	607 (58.3%)	76 (59.8%)
Twitter	203 (17.4%)	166 (15.9%)	37 (29.1%)
Snapchat	712 (60.9%)	631 (60.6%)	81 (63.8%)
Discord	453 (38.8%)	400 (38.4%)	53 (41.7%)
Twitch	276 (23.6%)	243 (23.3%)	33 (26%)
**Sporting activities (X**^**2**^ [4] **= 9.800)**			
never	403 (34.5%)	347 (33.3%)	56 (44.1%)
once a week	181 (15.5%)	162 (15.6%)	19 (15%)
twice a week	226 (19.3%)	200 (19.2%)	26 (20.5%)
three times a week	156 (13.4%)	143 (13.7%)	13 (10.2%)
more than three times a week	202 (17.3%)	189 (18.2%)	13 (10.2%)*

Logistic regression performed to ascertain the role of the above parameters were found the following to be significant in an univariate analysis: gender, age, place of stay, early exposure to internet, urgency to answer online, daily chat with friends, frequency of personal contacts, sporting habits, time spent online, daily time interval and online accounts among internet addicts and normal users. The logistic regression model was statistically significant (*p* = 0,008, X 2 = 21,0). The model explained 45.8% (Nagelkerke R 2) of variance in internet addiction and correctly classified 90.1% of cases.

Female sex (OR = 2.760, *p* = 0,006, CI 95% 0.065 to 0.384), younger age (11–12 years) (OR = 3.812, *p* < 0.001, 95% CI: 1.747–4.731), early exposure to internet (OR = 3.466, *p* = 0.001, 95% CI 1.535–5.446), living in a small village (OR = 1.081, *p* = 0,002, 95% CI 1.041–1.186) urgency to answer online (OR = 4.677, *p* = 0,000, 95% CI: 2.714–6.639), decreased frequency of personal contacts with friends (OR = 2.897, *p* = 0.004, 95% CI: 1.037–1.681) and spending more than 6 h online (OR = 12.913, *p* < 0.001, 95% CI: 10.798–14.892), morning and nighttime internet use (OR = 3.846, *p* = 0.000, CI 95% 1.886–5.810) and never doing any sports (OR = 2.016, *p* = 0.044, 95% CI: 1.050–3.354) were independently associated with problematic internet use (Table 4).

**Table 4:** Results of the multivariate analysis

*N* = 1168	B	SE B	p	OR	CI 95% Lower	CI 95% Upper
sex (female)*	0.224	0.810	0.006	2.760	1.747	4.731
age (11–12 years)*	0.134	0.035	0.000	3.812	3.539	5.746
place of stay*	0.114	0.037	0.002	1.081	1.041	1.186
start of internet use (5–6 years)*	0.070	0.020	0.001	3.466	1.535	5.446
urgency to answer*	0.398	0.085	0.000	4.677	2.714	6.639
daily chat with friends	0.006	0.36	0.872	0.162	0.948	2.025
rarely meeting with friends*	0.113	0.039	0.004	2.897	1.037	1.681
time spent online (more than 6 h)*	0.287	0.022	0.000	12.913	10.798	14.892
time of day online (night, morning)*	0.840	0.218	0.000	3.846	1.886	5.810
> 2 online accounts (Twitter, Youtube, TikTok)	2.939	1.511	0.535	0.621	2.027	3.905
never doing any sports*	0.160	0.054	0.044	2.016	1.050	3.354

## Discussion

Our study is among the first from Hungary and worldwide focusing on the prevalence and risk factors of PUI among elementary school children. The rate of problematic users was more than 10% in our study population which is comparable to the data presented in the literature [26–28, 31, 32]. Widely used questionnaires (for the detection of PUI) may not be suitable for this age group (10–14 years), therefore, we carried out an official translation and linguistic validation of a potentially more suitable questionnaire with the permission of the authors. The rate of PUI was twice as high as suggested by the original questionnaire nearly 20 years ago, which can be explained by the passing of time and the widespread use of (more affordable and fast) internet services [1, 27].

Slightly surprisingly, PUI was more common in females than males, which contradicts the findings of previous studies. Apart from gender differences, the goal of internet use can also be an important predictor of addiction for example males tend to spend more time with online gaming and cybersex while females appeared to show a greater tendency toward social media use [33–37]. In our study population among the goals of internet use, watching videos and having more online social media accounts were associated with PUI, and as social media use (and addiction) is dominated by females according to previous findings in the literature, therefore, this may explain the detected gender differences [33]. Interestingly, only female gender was an independently associated factor of PUI in a multivariate analysis in our study population, which is not a unique finding, as it has also been shown by several previous studies [34, 35].

Younger age (11–12 years) was also associated with PUI as well as early exposure to the internet. It is well-documented that pre-school internet use as well as the lack of proper parental control are predisposing factors of PUI [36–38]. The previously mentioned parameters were independent parameteres of PUI in both uni- and multivariate analysis emphasizing the importance of both parental and school prevention strategies to avoid later problematic use. Time spent online was also an important parameter of problematic use. Similar to our previous studies in adolescent and adult populations, spending 6 h or more online was an independently associated with the phenomenon, although, previous studies have suggested a 2- hour cut-off time interval [9, 11, 21, 39]. This time interval (being online less than 2 h) was found to be protective in our study, underlining the importance of proper control. The goals of internet use such as watching videos and having social media accounts were also strongly related to PUI, however, only in an univariate and not a multivariate analysis. It should be mentioned that both morning (which means schooltime) and nighttime (presumably at the expense of sleep) internet use were independently associated with the phenomenon in our study population, which is in line with previous studies also drawing attention to the importance of adequate control measures [9, 40].

PUI was previously shown to be common among people living in rural areas which may also reflect a lower socioeconomical status, the role of which, as an independent risk factor, was also confirmed in our study [9]. Lower parental education level was also related to this phenomenon, but could not be confirmed by our results. However, not knowing parents’ educational levels was a predisposing factor only in an univariate, but not in a multivariate analysis. We have no obvious explanation for this phenomenon, we speculate the lack of proper child-parent connections (possible neglect) as a potential background.

Urgency to answer online messages and connecting with friends mainly online at the expense of personal relationships were also related to PUI, furthermore, lack of participation in any form of any physical activity was also associated with problematic internet use in both univariate and multivariate analysis. The role of these social factors are rarely studied among problematic users, only a few studies have dealt with the topic before [34]. The lack of a person of trust to talk to as well as being online instead of meeting with close friends may increase the possibility of PUI [34, 39, 42]. Being physically active (doing sports etc.) may promote the formation of friendships and can be protective against PUI [34, 40, 41].

Having multiple accounts was also related to PUI. The minimum age at which one is allowed to create an internet account is 13 in most countries. Nevertheless, the vast majority of children aged under 13 had at least 2, but more commonly 3 or more internet accounts, which could be associated with problematic usage among those aged over 13 years as well. Internet use is poorly regulated and controlled by laws in the majority of countries, and our investigation draws attention to this being a potentially important risk factor [43].

In conclusion, our study is among the first to focus on the prevalence and risk factors of PUI among (Hungarian) elementary school children. In contrast to widely used questionnaires we have translated and adapted a questionnaire specifically designed for this age group into Hungarian. Based on our results, more than 10% suffered from PUI in our study population, which is a relatively high rate. Early exposure to the internet as well as younger age were strongly related to this phenomenon. Time spent online as well as the daily time interval of internet use are important predisposing factors. Being online at the expense of personal relationships and the lack of physical activity, social factors that have been rarely studied, are worth paying more attention to in order to prevent the development of problematic usage.

Our work has certain limitations. Problematic usage of the internet (or so called internet addiction) is still considered to be a phenomenon and not a medical condition, therefore, we have to deal with a lack of standardized methodology and thus that of objective measurement tools. Our study involved more than 1000 children, but as it was a cross-sectional research in nature, the sample is not representative, thus our findings cannot be generalized and conclusions are only limited to the current study population. Furthermore, our study contains children (aged 9) and adolescents (aged 16) and the age distribution shows considerable disproportions, which may sigificanty affects our findings. The fact that it was a self-completion questionnaire survey may have resulted in some degree of bias which may significantly have influenced our results. These limitations may have had a significant effect on our results and conclusions.

## Data Availability

The dataset supporting the conclusions of this article is available on request to the corresponding author.
